# A comparative field efficacy trial of three treatment programs against endo- and ectoparasites in naturally infected dogs

**DOI:** 10.3389/fvets.2024.1460452

**Published:** 2024-09-05

**Authors:** Cameron Raw, Rebecca J. Traub, Anke Wiethoelter

**Affiliations:** Melbourne Veterinary School, Faculty of Science, University of Melbourne, Parkville, VIC, Australia

**Keywords:** canine, ivermectin, moxidectin, oxibendazole, afoxolaner, flumethrin, hookworm

## Abstract

**Introduction:**

Tropical climates in remote Aboriginal and Torres Strait Islander communities in northern Australia are conducive to the transmission of canine helminths such as hookworms, as well as ectoparasites such as fleas and ticks. In addition to their veterinary importance, these parasites may present a zoonotic risk either directly, or as potential vectors for bacterial pathogens. These factors necessitate efficacious and effective antiparasitic treatment programs for community dogs.

**Methods:**

A cluster-randomised trial was performed on three islands in the Torres Strait to examine the short-term efficacy and medium-term effectiveness of three treatment programs. Treatments administered included oral oxibendazole/praziquantel (Paragard^®^) and oral afoxolaner (Nexgard^®^); topical moxidectin/imidacloprid (Advocate^®^) and imidacloprid/flumethrin collars (Seresto^®^); and off-label oral ivermectin (Bomectin^®^). Canine faecal samples were collected and examined for endoparasites by faecal flotation and real-time PCR at baseline, 7–11 days after treatment and 6 months later.

**Results:**

The proportion of dogs positive for *Ancylostoma caninum* at baseline and negative at day 7–11 was 9% (95% CI 4.4–17.4) for dogs treated with oxibendazole, 56.4% (95% CI 41–70.7) for moxidectin, and 89.7% (95% CI 73.6–96.4) for ivermectin. Faecal flotation results showed a greater than 90% egg reduction in 29.2% (95% CI 19.9–40.5) of dogs treated with oxibendazole, 79.4% (95% CI 63.2–89.7) for moxidectin, and 95% (95% CI 76.4–99.1) for off-label ivermectin. Elimination of ectoparasite infestation was observed at day 7–11 in 69.9% (95% CI 56.7–80.1) of dogs treated with afoxolaner, 80% (95% CI 60.9–91.1) with imidacloprid/flumethrin collars, and 0% (95% CI 0–11.7) for off-label ivermectin. Mixed effects modelling revealed only treatment group to be significantly associated with outcome measures.

**Discussion:**

Based on these study results, the poor efficacy of oxibendazole against *A. caninum* renders it inept for treatment, while ivermectin and moxidectin were suitable. Ivermectin was unsuitable for ectoparasite treatment due to its poor efficacy, while afoxolaner and imidacloprid/flumethrin collars appear suitable.

## 1 Introduction

In tropical climates, and particularly in remote community settings, canine endoparasites and ectoparasites and the diseases they vector cause significant morbidity and mortality in dogs and are also responsible for some of the most important and well recognised zoonoses affecting humans (1–4). Endoparasites such as hookworms of the genus *Ancylostoma* spp., threadworms (*Strongyloides* spp.) and roundworms (*Toxocara canis*) constitute some of the most prevalent canine zoonotic helminths of stray, semi-domesticated and pet dogs throughout tropical regions of the world (5, 6). Infections with these parasites can result in asymptomatic to serious clinical manifestations in dogs and people. For example, *Ancylostoma* spp. infections can cause profound haemorrhagic enteritis and anaemia in dogs, depending on parasite species and worm burden. *Ancylostoma* spp. infection in humans may cause cutaneous larva migrans, or in the case of *Ancylostoma caninum*, eosinophilic enterocolitis (5, 7). While most human intestinal infections with *A. caninum* were found to be caused by a single adult worm, more recent evidence suggests that patent infections are potentially possible (8). Infection with *Toxocara canis* may manifest as ocular toxocariasis with vision loss or retinal damage or as visceral toxocariasis with wheezing, asthma, fever, or abdominal pain (9).

High burdens of fleas (*Ctenocephalides felis*) and brown dog ticks (*Rhipicephalus linnaei*) in community dogs contribute to the spread of tick-borne diseases ehrlichiosis, hepatozoonosis, babesiosis and anaplasmosis, while fleas may pose a zoonotic risk for the transmission of bartonellosis and flea-borne spotted fever (10–13). In addition to the risk of vector-borne diseases, pruritis caused by even transient flea or tick infestations or bites may predispose humans to chronic secondary skin infections with potential sequelae of impetigo, rheumatic fever, or rheumatic heart disease (14, 15).

As in Aboriginal communities across other parts of Australia, dogs in Torres Strait Islander communities may have many different roles including companion, hunting partner, source of protection, or cultural or spiritual roles (16–18). These important roles, as well as the often free-roaming nature and large populations of dogs in these communities, may place community members at risk of acquiring parasite and flea-borne zoonotic pathogens either directly through close contact, or indirectly through contact with, or ingestion of parasitic stages in contaminated soil and bedding (19).

Efficacious endo- and ectoparasitic treatments are essential to mitigate the morbidity related to canine parasites. The remoteness of many Australian Aboriginal and Torres Strait Islander communities means that veterinary visits may be limited, sporadic or ultimately unattainable due to logistical or financial barriers. As such, identifying effective antiparasitic treatment programs which can be administered regularly without the need for veterinary oversight is of value to these communities. Off-label treatments require veterinary oversight to be administered as they are being used outside of the registered and labelled use (20). Such treatments have formed the mainstay of remote community veterinary antiparasitic treatment despite scarcity of evidence of their effectiveness in these settings. Evaluating the efficacy of off-label treatment is therefore of value, particularly to the veterinarians, local government departments or non-government organisations (NGOs) owing to their potential cost effectiveness (21, 22). With these factors in mind, the aim of this study is to examine the short-term efficacy and medium-term effectiveness of two labelled antiparasitic treatment programs in comparison to the off-label usage of ivermectin in a remote Torres Strait Islander community setting. The resulting evidence will inform antiparasitic programs which can be administered by community members either with or without veterinary oversight.

## 2 Materials and methods

### 2.1 Study setting and population

The Torres Strait Islands comprise over 270 small islands in the Torres Strait between the northernmost tip of mainland Australia in the state of Queensland and Papua New Guinea spanning an area of over 48,000 km^2^. Sitting at the border of equatorial savanna and monsoonal climate regions based on a modified Köppen climate classification system (23), the primary weather station for the islands recorded a mean annual rainfall of 1736 mm and mean temperature range of 24.7–30.5°C between 1995 and 2023 (24).

**Figure 1:**
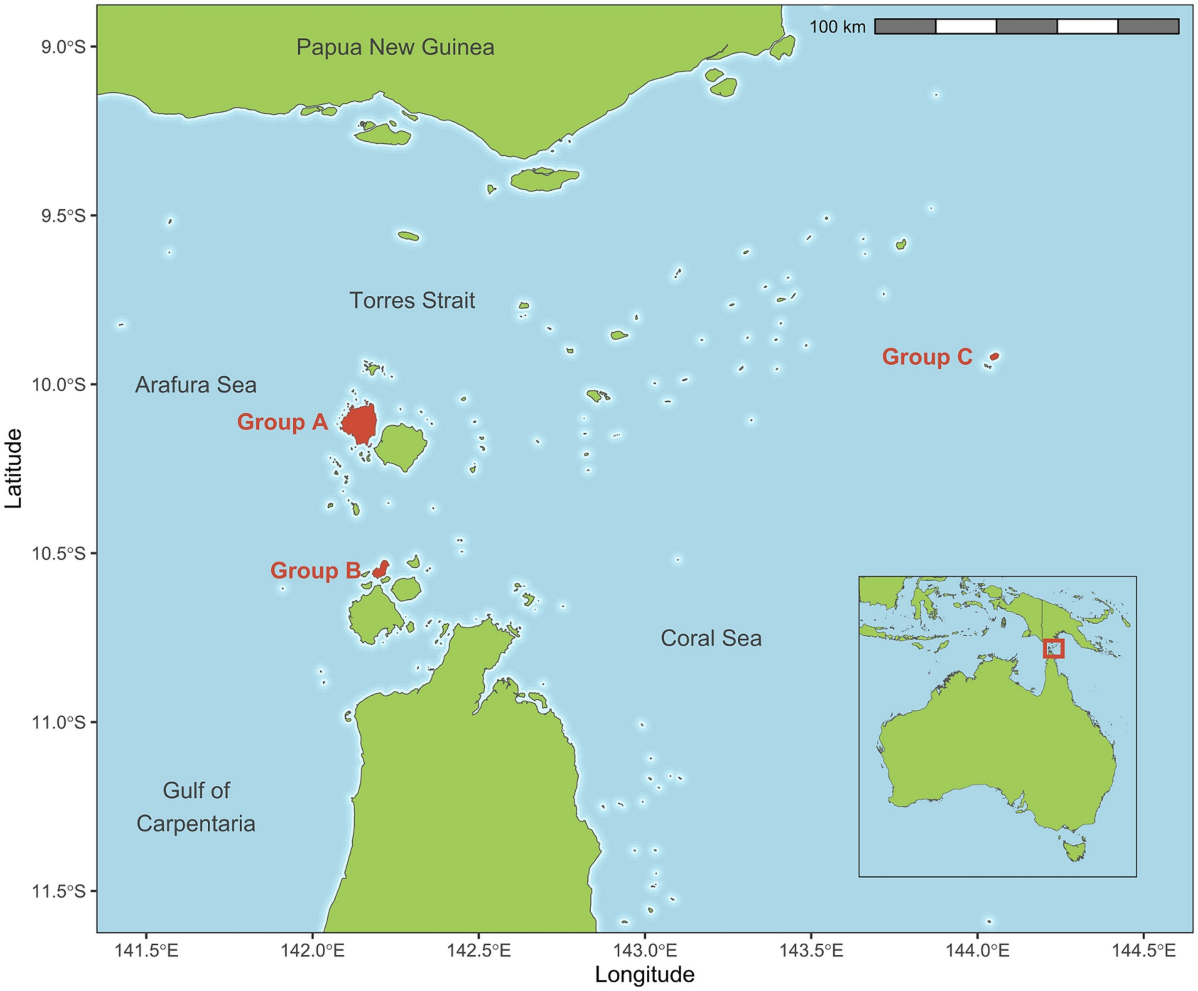
Torres Strait region map with shaded and labelled study islands.

Dogs on three remote islands were enrolled in this cluster-randomised trial. Islands were selected based on recommendations from the Torres Strait Islands Regional Council regarding adequate dog numbers present as well as community consultation and acceptance of the proposed study. Locations of the selected islands are shown in Figure 1. Torres Strait Islander community engagement and leadership was crucial to this study. In-person consultation was conducted with local Environmental Health Worker staff to ascertain what was important to the community and to develop a feasible study methodology. This was followed by consultation with elders and elected council representatives of all island groups regardless of their inclusion in the study, and approval of a formal research proposal. This study was also approved by the University of Melbourne Animal Ethics Committee (ID: 10298).

All dog owners on each selected island were approached to provide verbal and written consent to have their dogs recruited into the study. Dogs were not recruited if owners did not consent or were not present to provide consent. Dogs on each island were assigned to the same treatment arm to ensure consistent administration of ongoing treatments and to reduce the risk of environmental contamination influencing other treatment group outcome measures. Treatment arms consisted of; Group A—oral tablets administered at 22.5 mg oxibendazole/5 mg praziquantel per kilogram bodyweight (Paragard^®^, Boehringer Ingelheim) and oral chews administered at 2.5 mg afoxolaner per kilogram bodyweight (Nexgard^®^, Boehringer Ingelheim); Group B—topical 1% moxidectin/10% imidacloprid applied at 0.1 mL per kilogram bodyweight (Advocate^®^, Elanco) and a 10% imidacloprid/4.5% flumethrin polymer matrix collar (Seresto^®^, Elanco) administered according to the labelled instructions and; Group C—off-label oral ivermectin (Bomectin^®^, Elanco) administered at 200 μg/kg in bread with flavoured paste. As ivermectin administration in this context is off-label usage, it required oversight from a registered veterinary practitioner.

### 2.2 Data collection

At baseline, dog and owner names and address details were collected for the purpose of follow-up reidentification. Other dog details recorded at the time of enrolment included sex, sterilisation status, estimated weight, and age group. Age group information was provided by dog owners at the time of enrolment or was estimated by a veterinarian on examination of the dog. Age group classifications consisted of puppies which were less than 6 months old, young dogs which were 6 months to 2 years old, adults which were 2–8 years old, and old dogs which were greater than 8 years old. Any overt skin lesions were noted, and a targeted patch examination technique of predilection sites was used to establish a semi-quantitative measure of tick burden on each dog as described by Brianti et al. (25). Briefly, a tick score of zero indicates no ticks detected, a score of 1 indicates between 1 and 5 ticks detected, a score of 2 indicates 6–20 ticks detected, a score of 3 indicates 21–50 ticks detected, a score of 4 indicates 51–100 ticks detected and a score of 5 indicates over 100 ticks detected. The same system was employed to determine flea burden. Single faecal samples were collected from each dog rectally, or from the ground if rectal collection was not possible and a fresh ground sample identifiable to the dog was available. All faecal samples were immediately stored in DNA/RNA Shield (Zymo Research, Irvine, USA) at a 1:2 ratio for transport at room temperature to the University of Melbourne for laboratory analysis. At this point, treatments were administered per specified treatment arm and dogs remained under their owners’ care thereafter.

Follow-up sampling was conducted by the same method 7–11 days post-treatment. This timeframe allows detection of reduction or cure of initial infection whilst avoiding new or re-infections as it is shorter than the prepatent period of *Ancylostoma* spp. Dogs were reidentified from recorded data to allow comparison of baseline and post-treatment data. Repeat measures of flea and tick count were also recorded.

Dogs again remained in their owners’ care and were treated according to their treatment arm 3 months post-baseline. Treatments were administered by trained local Environmental Health Workers. Six months post-baseline, dogs were reidentified and underwent repeat faecal sampling and flea and tick counting.

### 2.3 Coproscopic and molecular methods

One gram of faeces was subjected to a quantitative faecal float using a centrifugal faecal flotation (CFF) method with saturated sodium chloride and sucrose (specific gravity 1.27). Parasite eggs were manually counted and converted to eggs per gram (EPG) by multiplying counts by the inverse of the faecal sediment measured in the centrifuge tube to allow sample comparison.

DNA was extracted from 200 mg of faeces of each sample using the Maxwell^®^ RSC PureFood GMO and Authentication Kit (Catalog no. AS1600, Promega Corporation, Madison, USA) with the Maxwell^®^ RSC 48 Instrument (Catalog no. AS8500, Promega Corporation, Madison, USA) using a modified method as described by Massetti et al. (26).

Extracted DNA was subjected to multiplex qPCR assays for the detection of four species of canine hookworm including *Ancylostoma caninum*, *Ancylostoma ceylanicum*, *Uncinaria stenocephala*, and *Ancylostoma braziliense* as well as *Strongyloides* spp. according to published protocols (26, 27). Internal amplification controls were performed using equine herpes virus (EHV4) primers (EHV-F, EHV-R), probe (EHV probe) and EHV4 synthetic DNA fragments containing the target sequence (gBlock^®^ Gene Fragments, IDT^®^ Technologies, Skokie, USA). DNA extraction controls were performed with mammalian primers (MAM-F, MAM-R) and probe (MAM probe) (27–29). Synthetic DNA fragments containing the target sequence of each parasite species (gBlock^®^ Gene Fragments, IDT^®^ Technologies, Skokie, USA) were used as positive controls and no-template negative controls were included in all runs. A five channel AriaMx Real-time PCR System (Agilent, Santa Clara, USA) was used for the amplification, detection, and data analysis of all samples (Agilent Aria software).

### 2.4 Statistical analysis

Demographic and physical examination and laboratory data were recorded on paper then transferred, cleaned, and validated in an electronic spreadsheet (Microsoft Excel v. 1908, Microsoft Corporation, Redlands, USA). Recoding of variables was conducted where necessary and data was analysed and plotted in R (v. 4.2.2) (30) using RStudio and contributed packages lme4 (v. 1.1–34) (31), emmeans (v. 1.8.7) (32), ggplot2 (v. 3.4.2) (33), epiR (v. 2.0.60) (34), and terra (v.1.7–55) (35). Flea and tick scores were combined to an ectoparasite score and subsequently used as a binary variable (present/absent) to account for low frequencies. Similarly, age group categories were collapsed to dogs under 1 year of age and dogs over 1 year of age to account for low frequencies.

Dog demographic data including age group, sex and desexed status as well as qPCR-based endoparasite prevalence, hookworm EPG distributions and ectoparasite prevalence were described for each treatment arm. Short-term data between baseline and day 7–11 post-treatment permitted the calculation of efficacy measures for each treatment; those being the performance of each treatment under close to ideal conditions which do not include new re-infections. Cure rates (CR) were calculated as a percentage in which the number of dogs qPCR-positive for a parasite species pre-treatment and negative 7–11 days post-treatment was divided by the total number of dogs positive for the parasite species pre-treatment. 95% confidence intervals were calculated for prevalence and CR estimates using the epi.conf function in the epiR package. Cure rates for ectoparasites were also conducted in the same manner for each treatment arm and demographic group.

For dogs testing positive for hookworm eggs at baseline, egg reduction rates (ERR) were calculated as a percentage, where the 7–11 days post-treatment count was subtracted from the baseline count and divided by the baseline count. Per World Association for the Advancement of Veterinary Parasitology (WAAVP) guidelines and International Co-operation on Harmonisation of Technical Requirements for Registration of Veterinary Medicinal Products (VICH) guidelines as adopted by the Australian Pesticides and Veterinary Medicines Authority (APVMA), a 90% ERR threshold was used to indicate an anthelmintic as efficacious for label claim requirements (36–38). In the present study, the proportion of dogs achieving a 90% or greater ERR in each treatment and demographic group was calculated along with 95% confidence intervals.

Considering all timepoint data up to 6 months permits the calculation of effectiveness measures which, in contrast to efficacy measures, are inclusive of real-world influences such as re-infection. Generalised linear mixed models were used to assess associations between treatment group and EPG and treatment group and ectoparasite infestation based on Poisson and binomial family models, respectively. Individual dog and island were included as random effects and age group, sex and desexed status were included as fixed effects. Backward stepwise variable selection was used to arrive at the final model considering a *p*-value of <0.05 significant. A data dispersion ratio was calculated from the sum of residual squares divided by the number of observations. R code for this analysis is included in Supplementary File S1.

## 3 Results

Treatment arms consisted of 80 dogs in Group A, 51 in Group B and 44 in Group C at baseline. Populations varied in each treatment group with respect to the representation of age group, sex and desexed status. Demographic data for dogs in each treatment group at baseline are shown in Table 1. Adult dogs were the largest age group in each treatment group followed by young dogs. No puppies were included in Group C. Proportions of male dogs were higher in Groups A and C, while more females were found in Group B. More desexed dogs were present in Group C while more entire dogs were present in Groups A and B. One dog in Group B was not present for resampling at post-treatment follow up and was therefore excluded from efficacy analysis. 18 dogs from Group A, 15 dogs from Group B and 20 dogs from Group C had either died or were not present for sampling at the six-month timepoint and were therefore excluded from medium-term effectiveness analysis. All other dogs were present for sampling at all time points.

**Table 1:** Dog demographic data from each treatment group.

Variable and category	Total *n* (%)	Group A *n* (%)	Group B *n* (%)	Group C *n* (%)
Age group				
Puppy	11 (6.3)	4 (5)	7 (13.7)	0 (0)
Young	45 (25.7)	18 (22.5)	10 (19.6)	17 (38.6)
Adult	103 (58.9)	53 (66.2)	32 (62.7)	18 (40.9)
Old	16 (9.1)	5 (6.2)	2 (3.9)	9 (20.5)
Sex				
Female	74 (42.3)	33 (41.2)	26 (51)	15 (34.1)
Male	101 (57.7)	47 (58.8)	25 (49)	29 (65.9)
Desexed				
Yes	69 (39.4)	27 (33.8)	19 (37.3)	23 (52.3)
No	106 (60.6)	53 (66.2)	32 (62.7)	21 (47.7)

**Figure 2:**
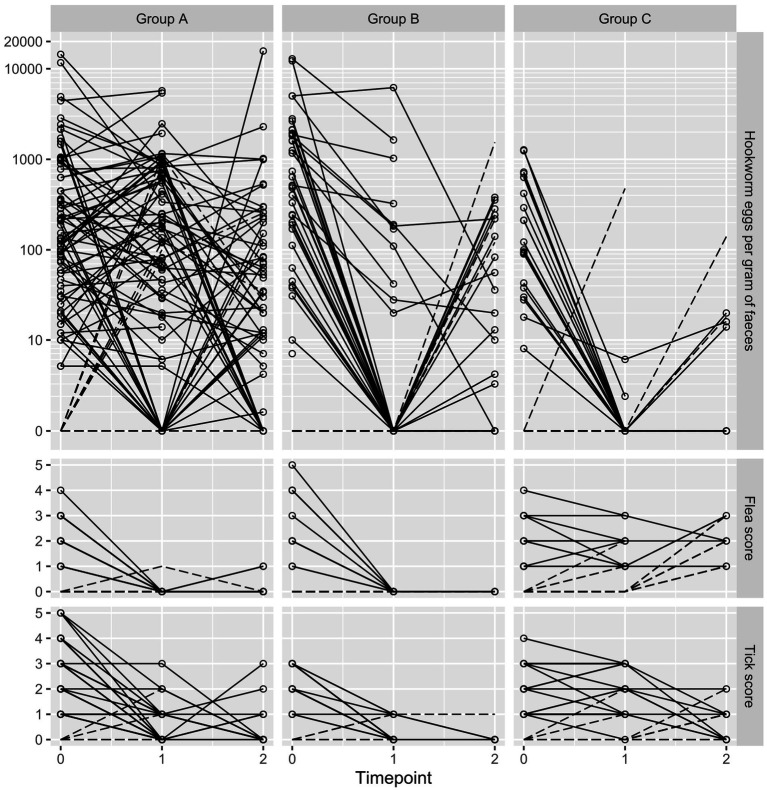
Trellis plot of hookworm eggs per gram, flea score and tick score at baseline (timepoint 0), 7–11 days following treatment (timepoint 1) and 6 months later (timepoint 2). Each line shows results of an individual dog. Dotted lines represent dogs with a baseline count or score of zero.

Mammalian DNA extraction controls were positive for all samples. Only *A. caninum* and *Strongyloides* spp. were detected by the multiplex qPCR and only *A. caninum* was detected at levels allowing for before-and-after comparison in individual dogs. Overall baseline qPCR-based prevalence of *A. caninum* was 83.9% (95% CI 77.7–88.6) with 97.5% (95% CI 91.3–99.3) in Group A, 78.4% (95% CI 65.4–87.5) in Group B and 65.9% (95% CI 51.1–78.1) in Group C. Baseline microscopy-based EPG varied widely, with a geometric mean of 219 (range 0–14,430) and high degrees of skewness (4.85) and kurtosis (26.04). Baseline EPG was highest in puppies, with three puppies (and a single adult) shedding more than 10,000 EPG. Individual dog *A. caninum* EPG counts, flea score and tick score at each time point are presented in Figure 2.

Cure rates and ERR results for dogs which tested positive to *A. caninum* via qPCR CFF and positive for ectoparasites via patch examination are shown in Table 2.

**Table 2:** Endoparasite and ectoparasite outcome measures at 7–11 days post-treatment by treatment and demographic group for dogs which were positive at baseline.

Variable and category	*A. caninum* qPCR cure rate		Dogs achieving *A. caninum* 90% egg reduction rate		Ectoparasite cure rate	
	*n*	% (95% CI)	*n*	% (95% CI)	*n*	% (95% CI)
Total	146	37.7 (30.2–45.8)	126	53.2 (44.5–61.7)	110	53.6 (44.4–62.7)
Treatment						
Group A	78	9 (4.4–17.4)	72	29.2 (19.9–40.5)	56	69.6 (56.7–80.1)
Group B	39	56.4 (41–70.7)	34	79.4 (63.2–89.7)	25	80 (60.9–91.1)
Group C	29	89.7 (73.6–96.4)	20	95 (76.4–99.1)	29	0 (0–11.7)
Age group						
Puppy	9	22.2 (6.3–54.7)	9	55.6 (26.7–81.1)	8	75 (40.9–92.9)
Young	38	47.4 (32.5–62.7)	32	46.9 (30.9–63.6)	27	40.7 (24.5–59.3)
Adult	89	32.6 (23.7–42.9)	78	55.1 (44.1–65.7)	64	60.9 (48.7–71.9)
Old	10	60 (31.3–83.2)	7	57.1 (25–84.2)	11	27.3 (9.7–56.6)
Sex						
Female	63	39.7 (28.5–52)	54	53.7 (40.6–66.3)	48	56.2 (42.3–69.3)
Male	83	36.1 (26.6–46.9)	72	52.8 (41.4–63.9)	62	51.6 (39.4–63.6)
Desexed						
Yes	54	44.4 (32–57.6)	44	48.8 (38.3–59.4)	38	44.7 (30.1–60.3)
No	92	33.7 (24.9–43.8)	82	61.4 (46.6–74.3)	72	58.3 (46.8–69)

Baseline prevalence for fleas was 37.5% (95% CI 27.7–48.5) for dogs in Group A, 23.5% (95% CI 14–36.8) for Group B and 36.4% (95% CI 23.8–51.1) for Group C. Baseline prevalence for ticks was 65% (95% CI 54.1–74.5) for dogs in Group A, 39.2% (95% CI 27–52.9) for Group B and 52.3% (95% CI 37.9–66.2) for Group C. Positive or negative ectoparasite status derived from this led to the calculation of cure rates presented in Table 2.

Coefficient estimates for the final EPG Poisson and ectoparasite infestation binomial models are presented in Table 3. Neither age group, sex nor desexed status were significantly associated with EPG or ectoparasite infestation between baseline and day 7–11 or between this time point and 6-months and were thus removed from the final model. Intraclass correlations were calculated, with greater than 99.9% of the variation in EPG and ectoparasite infestation attributable to differences between dogs, rather than island clusters. Overdispersion was present in the EPG model with a data dispersion ratio of 406.

**Table 3:** Mixed effects model outputs for associations with changes in eggs per gram of faeces and ectoparasite infestation.

		Association with *A. caninum* EPG of faeces			Association with ectoparasite infestation		
Variable	Category	Coefficient estimate	Standard error	*p-*value	Coefficient estimate	Standard error	*p-*value
Fixed effects							
**Treatment group**				<0.001*			<0.001*
	Group A	*Reference*			*Reference*		
	Group B	−0.90	0.54	0.097	−1.62	0.71	0.023
	Group C	−3.81	0.58	<0.001	−0.15	0.73	0.84
**Timepoint**				<0.001*			<0.001*
	Baseline	*Reference*			*Reference*		
	Day 7–11	−0.53	0.01	<0.001	−3.62	0.70	<0.001
	6 months	−0.54	0.01	<0.001	−5.23	0.94	<0.001
**Treatment** × **timepoint**				<0.001*			0.002*
	Group A × baseline	*Reference*			*Reference*		
	Group B × Day 7–11	−1.29	0.01	<0.001	0.21	0.86	0.803
	Group C × Day 7–11	−2.05	0.05	<0.001	3.81	0.96	<0.001
	Group B × 6 months	−1.22	0.02	<0.001	−0.08	1.54	0.957
	Group C × 6 months	−2.20	0.07	<0.001	24.2	209.02	0.908
Random effects							
	Individual dog variance	8.94			5.28		
	Island cluster variance	<0.001			<0.001		

* Variable level *p*-values were calculated using the joint_tests function from the emmeans package.

## 4 Discussion

This study found that the treatment administered to each animal group was the most significant factor associated with reductions in *A. caninum* egg shedding (EPG) as well as presence or absence of ectoparasite infestation. Results indicate that demographic variables of age group, sex and desexing status are not associated with anti-parasiticide efficacy and effectiveness in this setting.

For treatment of *A. caninum*, off-label ivermectin performed best in terms of both qPCR CR and 90% ERR. This supports the findings of a treatment trial by Bhanjadeo et al. (39), for which ivermectin administered at 200 μg/kg body weight to 12 dogs infected with *A. caninum* with a mean EPG of 1,725 at baseline, produced a CR and EPG reduction of 100% at day 15 post-treatment. Studies have also demonstrated high efficacy of ivermectin against *A. caninum* at doses as low as 10 μg/kg (40, 41). In Australia, administration of ivermectin in dogs at doses above 6 μg/kg body weight represents off-label use. The lack of registered treatments may be partly due to the presence of the ABCB1 gene mutation often present in collie breeds and their crosses, which makes them more sensitive to toxic effects of ivermectin at doses used to target gastrointestinal helminths (42). In the author’s experience, the dogs living in remote Aboriginal and Torres Strait Islander communities tend to be medium-sized crossbreeds often known as ‘Australian camp dogs,’ and very rarely include collie dog genetics. The risk for these dogs is low and testing for gene mutations is not necessary (43). Nonetheless, care must be taken in populations which may have the ABCB1 gene mutation. Off-label drugs cannot be purchased by dog owners and require veterinary oversight, which comes at greater cost either at an individual dog level or in community-level animal health programs. Off-label usage also means that there may be less standardisation in the method and dose administered compared to commercially produced animal treatments, especially oral treatments. In this study, ivermectin was soaked into bread and covered with peanut butter for palatability, which was well accepted by the dogs, though palatability is often a challenge in these settings. However, acceptance cannot always be relied upon for any oral treatment in any dog whether it be commercially available or off-label.

While a study by Hellmann et al. (44) of the efficacy of topical moxidectin/imidacloprid in 131 naturally hookworm infected dogs found a geometric mean ERR of 99.92% 8 to 13 days following treatment, the proportion (29.2%) of dogs achieving a 90% ERR in the present study and low CR of 56.4% did not support this treatment’s efficacy to the same degree. One possible explanation for the reduced efficacy may be the inability to control for the application of the product to a dry coat and the avoidance of wetting the coat within 24 hours of application (45). Since dogs could not be supervised after treatment, it is possible that the rapid skin absorption of the moxidectin component of the product may have been disrupted. Moreover, individual clearance of moxidectin from the system may vary between dogs of different body condition score owing to their differing levels of adipose tissue, though this would be more relevant to moxidectin’s sustained larvicidal effect than its immediate adulticidal efficacy (46). Furthermore, differences in the distribution of body condition score did not differ significantly between treatment groups and would not sufficiently explain any differences in observed treatment effects.

Efficacy of oxibendazole based on this study was demonstrated to be poor against *A. caninum*. While tableting of dogs is the most difficult of the three endoparasitic treatments to administer in this study and is generally prone to failure due to dogs not accepting tablets, these treatments were all administered by a trained, registered veterinarian and all treatments were confirmed to have been swallowed. Individual dog data in Figure 2 shows multiple cases in which dogs were not only without cure or egg reduction but appear to have increases in egg counts following treatment. Several confounding factors are known to influence successive faecal egg counts in the same individual such as time of sampling, faecal consistency, and host diet (47). These effects may have been masked in the other treatment groups by treatment effects but were more evident in Group A due to a lack of efficacy.

Only a single study is known to have examined the efficacy of oxibendazole against hookworms in dogs. In this study, oral oxibendazole at a dose rate of 15 mg/kg administered to naturally infected dogs found a 94.6% reduction of *A. caninum* based on the reduction in the arithmetic mean EPG from baseline to 8–10 days post-treatment (48). The finding of such a high arithmetic mean ERR is surprising compared to the findings of the present study which used a higher dose rate of 22.5 mg/kg. The fact that only 11 dogs were initially infected with *A. caninum* in the Overgaauw and Boersema study, along with a lack of reported confidence intervals and accurate demographic data calls the validity of the presented results into question. Poor efficacy of oxibendazole, as with other benzimidazoles, may be related to its low aqueous solubility further compounded by the relatively rapid gut transit times of dogs (49). For that reason, efficacy of benzimidazoles is predominately time- rather than dose-dependent, with optimal efficacy typically only seen after repeated doses over 3–5 days (50). By contrast, in a recent study involving the development of an *in vitro* egg hatching assay to determine the ovicidal effects of anthelmintics it was revealed that oxibendazole, despite its poor adulticidal and larvicidal properties, demonstrated high potency against hookworm eggs, while eggs exposed to moxidectin or ivermectin showed relatively unchanged levels of maturation and hatching (51). This may point to a potential use for benzimidazoles in combination with an efficacious adulticidal and larvicidal treatment to immediately reduce environmental shedding of viable eggs, though further *in vivo* studies are necessary. Further studies are also required to investigate the potential for resistance to benzimidazole anthelminthics, and indeed all anthelmintics used for mass drug administration to treat *A. caninum*, especially with mounting evidence of β-tubulin gene fenbendazole resistance in this species (42).

Analysis of ectoparasite cure rates found oral afoxolaner given to Group A and imidacloprid/flumethrin collars given to Group B to be highly efficacious, which supports the findings of previous studies by Brianti et al. (25) and Fankhauser et al. (52). While the product label of Advocate^®^ and Seresto^®^ state that the products are still efficacious against ectoparasites after swimming, free-roaming dogs in these island settings frequently swim in salt water. Nevertheless, regular wetting of the coat did not appear to reduce efficacy of the imidacloprid and flumethrin concentrations within the coat in the hours to days after application in this study. Group C demonstrated very poor ectoparasitic efficacy, and although macrocyclic lactones are known to have lethal paralytic effects on arthropods at the time of exposure, this could not be observed at the time of follow-up and either the same or new flea and tick burdens were observed (50).

Random effects variance for island clusters in mixed effects modelling in this study was very low. While a lack of treatment randomisation would ordinarily be a limitation in many treatment trials, here it was a necessary study design feature. The impact of mass treatments was being assessed on a community, rather than individual animal level, including the ability of mass treatment to reduce environmental shedding and in turn re-infection rates. Had dogs been randomly allocated on each island, treatments with poor efficacy could have led to greater environmental contamination with parasites and greater chances of reinfection for all dogs over time, which may have reduced apparent medium-term effectiveness for what were otherwise more effective treatments. Realistically, differences in location in terms of veterinary and owner care would have been negligible and given that the time between pre-treatment and post-treatment sampling was insufficient to allow new patent reinfections, any differences based on location would have been minimal.

Random effects variance for individual dogs was, in comparison to island clusters, much higher. To allow maximal inclusion of dogs from areas with limited populations for the sake of statistical power, all dogs from all demographics were enrolled. Ideally at least 80 dogs would have been included in each treatment arm with a more equal distribution of age groups. While attempts were made to choose islands with the largest dog populations, a wave of parvovirus in the study islands leading to the deaths of several dogs immediately prior to initial sampling precluded reaching the planned sample size. Low numbers of dogs in the puppy and old age categories meant that collapsing these categories was necessary and that more detailed examination of age group associations with changes in outcome were not possible in mixed effects modelling. While puppies had the highest baseline EPG, it is biologically doubtful that age group alone would affect the clearance of infection holding all other variables constant. Other factors and comorbidities affecting young or old animals may affect their susceptibility to infection, however.

The Poisson model showed a high degree of overdispersion, which may be expected from field based faecal egg count data in which a large proportion of counts were zero along with some counts above 14,000 EPG. This overdispersion made for challenging model selection and meant that model fit parameters remained imperfect, even when other distributional assumptions were used. The presented final model selection and structure, however, is sufficient to demonstrate that associations between treatment and EPG or ectoparasite infestation were significant and that associations with demographic factors and cluster groups were not.

Access to efficacious and effective antiparasitic treatments is important in any setting, but particularly in remote Aboriginal and Torres Strait Islander communities where access to veterinary care and animal health products can have additional barriers and where the potential risks of zoonotic disease are especially relevant. The results of this study demonstrate that single-dose oxibendazole/praziquantel (Paragard^®^) has poor efficacy against the zoonotic dog hookworm *A. caninum*, while moxidectin/imidacloprid (Advocate^®^) and off-label ivermectin at 200 μg/kg appear efficacious. Furthermore, afoxolaner chews (Nexgard^®^) and imidacloprid/flumethrin collars (Seresto^®^) are efficacious against flea and tick infestation and may aid in preventing the spread of vector-borne diseases.

With the benefit of up-to-date efficacy data relevant to remote community field sites, local organisations can make informed decisions to help develop effective One Health programs and manage the risks of parasitic disease for all human and animal community members.

## Data Availability

The raw data supporting the conclusions of this article will be made available by the authors, without undue reservation.

## References

[1] Raw C Traub RJ Zendejas-Heredia PA Stevenson M Wiethoelter A. A systematic review and meta-analysis of human and zoonotic dog soil-transmitted helminth infections in Australian Indigenous communities. PLoS Negl Trop Dis. (2022) 16:e0010895. doi: 10.1371/journal.pntd.0010895, PMID: 36279298 PMC9632820

[2] Baneth G Thamsborg SM Otranto D Guillot J Blaga R Deplazes P. Major parasitic zoonoses associated with dogs and cats in Europe. J Comp Pathol. (2016) 155:S54–74. doi: 10.1016/j.jcpa.2015.10.179, PMID: 26687277

[3] Chen J Xu MJ Zhou DH Song HQ Wang CR Zhu XQ. Canine and feline parasitic zoonoses in China. Parasit Vectors. (2012) 5:1–8. doi: 10.1186/1756-3305-5-152, PMID: 22839365 PMC3431282

[4] Traub RJ Irwin P Dantas-Torres F Tort GP Labarthe NV Inpankaew T. Toward the formation of a companion animal parasite Council for the Tropics (CAPCT). Parasit Vectors. (2015) 8:271–5. doi: 10.1186/s13071-015-0884-4, PMID: 25963851 PMC4432817

[5] Bradbury R Traub RJ. Hookworm infection in Oceania. In: Loukas A, editor. Neglected tropical diseases - Oceania. Cham: Springer (2016).

[6] Page W Shield J O’Donahoo F Miller A Judd J Speare R. Strongyloidiasis in Oceania. In: Loukas A, editor. *Neglected tropical diseases - Oceania*. Cham: Springer (2016).

[7] Traub RJ Zendejas-Heredia PA Massetti L Colella V. Zoonotic hookworms of dogs and cats – lessons from the past to inform current knowledge and future directions of research. Int J Parasitol. (2021) 51:1233–41. doi: 10.1016/j.ijpara.2021.10.005, PMID: 34748782

[8] Ngcamphalala PI Lamb J Mukaratirwa S. Molecular identification of hookworm isolates from stray dogs, humans and selected wildlife from South Africa. J Helminthol. (2020) 94:e39, 1–9. doi: 10.1017/S0022149X19000130 30789121

[9] Chen J Liu Q Liu GH Bin ZW Hong SJ Sugiyama H. Toxocariasis: a silent threat with a progressive public health impact. Infect Dis Poverty. (2018) 7:59. doi: 10.1186/s40249-018-0437-0, PMID: 29895324 PMC5998503

[10] Barrs VR Beatty JA Wilson BJ Evans N Gowan R Baral RM. Prevalence of Bartonella species, *Rickettsia felis*, haemoplasmas and the Ehrlichia group in the blood of cats and fleas in eastern Australia. Aust Vet J. (2010) 88:160–5. doi: 10.1111/j.1751-0813.2010.00569.x, PMID: 20529020

[11] Dantas-Torres F. Biology and ecology of the brown dog tick, *Rhipicephalus sanguineus*. Parasit Vectors. (2010) 3:1–11. doi: 10.1186/1756-3305-3-26/FIGURES/8 20377860 PMC2857863

[12] Teoh YT Hii SF Graves S Rees R Stenos J Traub RJ. The epidemiology of *Rickettsia felis* infecting fleas of companion animals in eastern Australia. Parasit Vectors. (2018) 11:1–8. doi: 10.1186/S13071-018-2737-4/TABLES/3 29554953 PMC5859732

[13] Teoh YT Hii SF Graves S Rees R Stenos J Traub RJ. Evidence of exposure to *Rickettsia felis* in Australian patients. One Health. (2016) 2:95–8. doi: 10.1016/j.onehlt.2016.06.001, PMID: 28616481 PMC5441329

[14] O’Donel Alexander J. Flea bites and other diseases caused by fleas In: Arthropods and human skin. London: Springer (1984).

[15] Elliot AJ Cross KW Smith GE Burgess IF Fleming DM. The association between impetigo, insect bites and air temperature: a retrospective 5-year study (1999–2003) using morbidity data collected from a sentinel general practice network database. Fam Pract. (2006) 23:490–6. doi: 10.1093/FAMPRA/CML042, PMID: 16873392

[16] Smith BP Litchfield CA. A review of the relationship between Indigenous Australians, dingoes (*Canis dingo*) and domestic dogs (*Canis familiaris*). Anthrozoös. (2009) 22:111–28. doi: 10.2752/175303709X434149

[17] Constable S Dixon R Dixon R. For the love of dog: the human-dog bond in rural and remote Australian Indigenous communities. Anthrozoös. (2010) 23:337–49. doi: 10.2752/175303710X12750451259336

[18] Senior K Chenhall R McRae-Williams E Daniels D Rogers K. Dogs and people in Aboriginal communities: exploring the relationship within the context of the social determinants of health. Environ Health. (2006) 6:39–46.

[19] Smout F Schrieber L Speare R Skerratt LF. More bark than bite: comparative studies are needed to determine the importance of canine zoonoses in Aboriginal communities. A critical review of published research. Zoonoses Public Health. (2017) 64:495–504. doi: 10.1111/zph.12354, PMID: 28342271 PMC7159129

[20] Department of Agriculture Forestry and Fisheries. Agricultural and veterinary chemicals code (application requirements) instrument. (2014). Available at: https://www.legislation.gov.au/Details/F2020C00810 (Accessed September 20, 2023).

[21] Wilks K Williamson P. The dog health program in Aboriginal communities - a method for dog management in remote Aboriginal communities. Urban Anim Manag Conf. (1998)

[22] Bradbury L Corlette S. Dog health program in Numbulwar, a remote Aboriginal community in East Arnhem Land. Aust Vet J. (2006) 84:317–20. doi: 10.1111/j.1751-0813.2006.00028.x, PMID: 16958628

[23] Stern H De Hoedt G Ernst J. Objective classification of Australian climates. Aust Meteorol Mag. (2000) 49:87–96.

[24] Bureau of Meteorology. Horn Island climate statistics. (2023). Available at: http://www.bom.gov.au/climate/averages/tables/cw_027058.shtml (Accessed December 15, 2023).

[25] Brianti E Falsone L Napoli E Prudente C Gaglio G Giannetto S. Efficacy of a combination of 10% imidacloprid and 4.5% flumethrin (Seresto®) in slow release collars to control ticks and fleas in highly infested dog communities. Parasit Vectors. (2013) 6:210. doi: 10.1186/1756-3305-6-210, PMID: 23866926 PMC3728067

[26] Massetti L Wiethoelter A McDonagh P Rae L Marwedel L Beugnet F. Faecal prevalence, distribution and risk factors associated with canine soil-transmitted helminths contaminating urban parks across Australia. Int J Parasitol. (2022) 52:637–46. doi: 10.1016/j.ijpara.2022.08.001, PMID: 36007621

[27] Massetti L Colella V Zendejas PA Ng-Nguyen D Harriott L Marwedel L. High-throughput multiplex qPCRs for the surveillance of zoonotic species of canine hookworms. PLoS Negl Trop Dis. (2020) 14:e0008392. doi: 10.1371/journal.pntd.0008392, PMID: 32542036 PMC7316352

[28] Hii SF Senevirathna D Llewellyn S Inpankaew T Odermatt P Khieu V. Development and evaluation of a multiplex quantitative real-time polymerase chain reaction for hookworm species in human stool. Am J Trop Med Hyg. (2018) 99:1186–93. doi: 10.4269/ajtmh.18-0276, PMID: 30226132 PMC6221243

[29] Bialasiewicz S Whiley DM Buhrer-Skinner M Bautista C Barker K Aitken S. A novel gel-based method for self-collection and ambient temperature postal transport of urine for PCR detection of *Chlamydia trachomatis*. Sex Transm Infect. (2009) 85:102–5. doi: 10.1136/sti.2008.032607, PMID: 19004866

[30] R Core Team. R: A language and environment for statistical computing. Vienna, Austria: R Foundation for Statistical Computing. (2022). Available at: https://www.R-project.org/ (Accessed December 15, 2023).

[31] Bates D Maechler M Bolker B Walker S. Fitting linear mixed-effects models using lme4. J Statistical Software (2015) 67:1–48. doi: 10.18637/jss.v067.i01, PMID: 12928883

[32] Lenth R. emmeans: Estimated Marginal Means, aka Least-Squares Means. R package version 1.8.7. (2023). Available at: https://CRAN.R-project.org/package=emmeans (Accessed December 15, 2023).

[33] Wickham H. ggplot2: Elegant Graphics for Data Analysis. New York: Springer-Verlag (2016).

[34] Stevenson M Nunes ESwcf T Heuer C Marshall J Sanchez J Thornton R. epiR: Tools for the Analysis of Epidemiological Data. R package version 2.0.60. (2023). Available at: URL: https://CRAN.R-project.org/package=epiR (Accessed December 15, 2023)., PMID: 19004866

[35] Hijmans R. terra: Spatial Data Analysis. R package version 1.7-55. (2023). Available at: https://CRAN.R-project.org/package=terra (Accessed December 15, 2023).

[36] Geurden T Smith ER Vercruysse J Yazwinski T Settje T Nielsen MK. World association for the advancement of veterinary parasitology (WAAVP) guideline for the evaluation of the efficacy of anthelmintics in food-producing and companion animals: general guidelines. Vet Parasitol. (2022) 304:109698. doi: 10.1016/j.vetpar.2022.109698, PMID: 35305843

[37] Vercruysse J Holdsworth P Letonja T Conder G Hamamoto K Okano K. International harmonisation of anthelmintic efficacy guidelines (part 2). Vet Parasitol. (2002) 103:277–97. doi: 10.1016/S0304-4017(01)00615-X, PMID: 11777607

[38] Beugnet F Taweethavonsawat P Traversa D Fourie J McCall J Tielemans E. World Association for the Advancement of veterinary parasitology (WAAVP): second edition of guidelines for evaluating the efficacy of anthelmintics for dogs and cats. Vet Parasitol. (2022) 312:109815. doi: 10.1016/j.vetpar.2022.109815, PMID: 36335831

[39] Bhanjadeo R Patra RC Panda D Sahoo R Das DP Mohanty BN. Comparative efficacy of ivermectin and fenbendazole against ancylostomiasis in dogs. J Parasit Dis. (2022) 1:1–9. doi: 10.1007/S12639-022-01536-9/TABLES/4 PMC9998807 36910310

[40] Wang CI Huang XX Zhang YQ Yen QY Wen Y. Efficacy of ivermectin in hookworms as examined in Ancylostoma caninum infections. J Parasitol. (1989) 75:373–7. doi: 10.2307/3282591 2723924

[41] Daurio CP Roberson EL Seward RL. Efficacy of ivermectin in a beef-based chewable formulation against Ancylostoma caninum and Uncinaria stenocephala in dogs. J Parasitol. (1993) 79:768–70. doi: 10.2307/3283618 8410551

[42] Marsh AE Lakritz J. Reflecting on the past and fast forwarding to present day anthelmintic resistant Ancylostoma caninum–a critical issue we neglected to forecast. Int J Parasitol Drugs Drug Resist. (2023) 22:36–43. doi: 10.1016/J.IJPDDR.2023.04.003, PMID: 37229949 PMC10229760

[43] Beckers E Casselman I Soudant E Daminet S Paepe D Peelman L. The prevalence of the ABCB1-1Δ variant in a clinical veterinary setting: the risk of not genotyping. PLoS One. (2022) 17:e0273706. doi: 10.1371/JOURNAL.PONE.0273706, PMID: 36037240 PMC9423603

[44] Hellmann K Knoppe T Radeloff I Heine J. The anthelmintic efficacy and the safety of a combination of Imidacloprid and Moxidectin spot-on in cats and dogs under field conditions in Europe. Parasitol Res. (2003) 90:S142–3. doi: 10.1007/s00436-003-0919-1, PMID: 12928883

[45] Australian Pesticides and Veterinary Medicines Authority. Advocate for dogs APVMA approval 55321/131617. (2003). Available at: https://elabels.apvma.gov.au/55321ELBL.pdf (Accessed December 3, 2023).

[46] Bousquet-Mélou A Lespine A Sutra JF Bargues I Toutain PL. A large impact of obesity on the disposition of Ivermectin, Moxidectin and Eprinomectin in a canine model: relevance for COVID-19 patients. Front Pharmacol. (2021) 12:666348. doi: 10.3389/fphar.2021.666348 34093195 PMC8173197

[47] Morgan ER Lanusse C Rinaldi L Charlier J Vercruysse J. Confounding factors affecting faecal egg count reduction as a measure of anthelmintic efficacy. Parasite. (2022) 29:20. doi: 10.1051/parasite/2022017, PMID: 35389336 PMC8988865

[48] Overgaauw PAM Boersema JH. Anthelmintic efficacy of oxibendazole against some important nematodes in dogs and cats. Vet Q. (1998) 20:69–72. doi: 10.1080/01652176.1998.9694842, PMID: 9563164

[49] Sánchez S Sallovitz J Savio E Mckellar Q Lanusse C. Comparative availability of two oral dosage forms of albendazole in dogs. Vet J. (2000) 160:153–6. doi: 10.1053/tvjl.2000.0484 10985808

[50] Page SW. Chapter 10 - Antiparasitic drugs. In: JE Maddison, SW Page, DB Church, editors. Small Animal Clinical Pharmacology (Second Edition), W.B. Saunders, 2008. (2008) p. 198–260.

[51] Easland E Biendl S Keiser J. Development of a hookworm egg hatching assay to determine the ovicidal effects of anthelminthics. Parasit Vectors. (2023) 16:1–9. doi: 10.1186/S13071-023-05771-8 37143169 PMC10161531

[52] Fankhauser R Hamel D Dorr P Reinemeyer CR Crafford D Bowman DD. Efficacy of oral afoxolaner plus milbemycin oxime chewables against induced gastrointestinal nematode infections in dogs. Vet Parasitol. (2016) 225:117–22. doi: 10.1016/j.vetpar.2016.06.003, PMID: 27369586

