# Supplementary material for: A comparative field efficacy trial of three treatment programs against endo- and ectoparasites in naturally infected dogs
Source: Front Vet Sci. 2024 Sep 5;11:1460452. doi: 10.3389/fvets.2024.1460452 (PMC11410704; doi:10.3389/fvets.2024.1460452)
Supplement: Supplementary file 1 [file Data_Sheet_1.DOCX]

library(tidyverse)

library(lme4)

library(readxl)

library(emmeans)

library(performance)

library(broom.mixed)

library(lmerTest)

library(blmeco)

install.packages('epiDisplay')

# Load and define variables

directory <- "C:/Users/..." #Insert directory location of input files

dat_long <- read_excel(paste0(directory,"TSI_treatment_trial_project_data_long.xlsx"))

dat_wide <- read_excel(paste0(directory,"TSI_treatment_trial_project_data_wide.xlsx"))

dat_long <- dat_long %>% mutate(treatment = factor(as.numeric(factor(island))),

time_point = factor(time_point),

flea_yn = as.numeric(flea_count > 0), # Fleas present y/n

tick_yn = as.numeric(tick_count > 0), # Ticks present y/n

ecto_score = pmax(flea_count, tick_count), # Combining flea and tick score

ecto_yn = as.numeric(ecto_score > 0), # Any ectoparasites present y/n

age_collapsed = ifelse(age_group == "Puppy" | age_group == "Young", "Juvenile", "Adult or Old")) # Collapsing age categories

dat_wide <- dat_wide %>% mutate(treatment = factor(as.numeric(factor(island))),

qpcr_cure = t0_qpcr_result_ancylostoma_caninum - t1_qpcr_result_ancylostoma_caninum,

qpcr_cure = ifelse(qpcr_cure < 0, 0, qpcr_cure),

epg_perc = ((t0_epg_hookworm - t1_epg_hookworm)/t0_epg_hookworm) * 100,

epg_perc = ifelse(epg_perc < 0 | is.na(epg_perc), 0, epg_perc),

epg_90_perc_yn = as.numeric(epg_perc > 90),

flea_cure = ifelse(t0_flea_count > 0 & t1_flea_count == 0, 1,0),

tick_cure = ifelse(t0_tick_count > 0 & t1_tick_count == 0, 1,0),

t0_ecto_score = pmax(t0_flea_count, t0_tick_count),

t1_ecto_score = pmax(t1_flea_count, t1_tick_count),

ecto_cure = ifelse(t0_ecto_score > 0 & t1_ecto_score == 0, 1,0))

# Overdispersion function

overdisp_fun <- function(model) {

rdf <- df.residual(model)

rp <- residuals(model,type="pearson")

Pearson.chisq <- sum(rp^2)

prat <- Pearson.chisq/rdf

pval <- pchisq(Pearson.chisq, df=rdf, lower.tail=FALSE)

c(chisq=Pearson.chisq,ratio=prat,rdf=rdf,p=pval)

}

# Variance partition coefficient function

VPC <- function(model) {

VPC <- VarCorr(m1) %>%

as_data_frame() %>%

mutate(icc=vcov/sum(vcov))

VPC[,c(1,6)]

}

# ----------------------------------------------------------------------------------------------

## EPG hookworm

# Poisson final mixed effects model of effects on hookworm eggs per gram of faeces

m1 <- glmer(epg_hookworm ~ treatment*time_point + (1|island) +(1|dog_id) , family=poisson, data=dat_long)

summary(m1)

overdisp_fun(m1)

VPC(m1)

model_performance(m1)

joint_tests(m1)

emm1 <- emmeans(m1, specs="time_point", by="treatment")

emm1

pairs(emm1, adjust="none")

confint(pairs(emm1, adjust="none"))

# -----------------------------------------------

# Binomial final mixed effects model of effects on ectoparasite presence

m1_ecto <- glmer(ecto_yn ~ treatment*time_point + (1|island) +(1|dog_id) , family=binomial, data=dat_long)

summary(m1_ecto)

VPC(m1_ecto)

model_performance(m1_ecto)

joint_tests(m1_ecto)

emm1 <- emmeans(m1_ecto, specs="time_point", by="treatment")

emm1

pairs(emm1, adjust="none")

confint(pairs(emm1, adjust="none"))
